# 肺癌日间化疗的安全性分析

**DOI:** 10.3779/j.issn.1009-3419.2010.07.10

**Published:** 2010-07-20

**Authors:** 运 冯, 舜 陆, 正波 宋, 蕾 徐, 旖 施, 智伟 陈

**Affiliations:** 1 200030 上海，上海交通大学附属胸科医院院长办公室 Department of Deanery, Shanghai Chest Hospital, Shanghai 200030, China; 2 200030 上海，上海交通大学附属胸科医院肺部肿瘤临床医学中心 Shanghai Lung Tumor Clinical Medical Center, Shanghai Chest Hospital, Shanghai 200030, China; 3 200030 上海，上海交通大学附属胸科医院科教科 Department of Science and Education, Shanghai Chest Hospital, Shanghai 200030, China; 4 200030 上海，上海交通大学附属胸科医院医务科 Department of Medical, Shanghai Chest Hospital, Shanghai 200030, China

**Keywords:** 肺肿瘤, 日间化疗, 安全性, Lung neoplasms, Ambulatory chemotherapy, Safety

## Abstract

**背景与目的:**

日间化疗因具经济成本低、毒副反应不高的特点成为发达国家普遍采用的一种化疗模式，本文探讨上海市胸科医院日间化疗模式的可行性及安全性，为今后日间化疗模式的推广提供借鉴。

**方法:**

对所有入住日间化疗病房和普通化疗病房患者进行统计和记录，主要内容包括患者一般状况、化疗次数、病理类型、住院期间毒副反应、出院后出现的毒副反应。

**结果:**

2008年10月-2009年4月，我院日间病房共收治化疗患者416人次，普通病房共收治患者1 157人次，两组化疗的毒副反应总体差异不大，日间化疗患者的Ⅱ度以上胃肠道反应（21.88%）较住院患者低（38.89%）（*P* < 0.01），白细胞降低比例（49.28%）较住院患者（35.0%）为多（*P* < 0.01），日间化疗病房住院天数缩短（4.48天*vs* 14.04天，*P* < 0.01），医疗支出也大大降低（6 911.32元*vs* 14 623.59元，*P* < 0.01）。

**结论:**

日间化疗的住院时间和费用缩短，主要毒副反应为白细胞降低，化疗的累积毒性是其主要原因，需要引起注意，其它毒副反应未见明显差异，日间化疗是一种值得推广和借鉴的方式。

## Abstract (en)

**Background and objective:**

Ambulatory chemotherapy has now been a popular way of treatment in developed countries, for its less medical costs and incidence of adverse events. The aim of this study is to estimate the feasibility and safety of ambulatory chemotherapy in lung cancer in Shanghai chest hospital.

**Methods:**

Medical data including performance status, cycles of chemotherapy, pathologic type, adverse events in hospital and adverse events after discharge from hospital.

**Results:**

We estimated a total of 1 573 lung cancer patients admitted in Shanghai chest hospital, during October 2008 to April 2009, including 416 patients treated in the way of ambulatory chemotherapy, 1 157 patients treated in the routine way. Rate of grade Ⅲ/Ⅳ gastrointestinal toxicity was fewer in the ambulatory chemotherapy group (21.88%) than routine chemotherapy group (*P* < 0.01); meanwhile, rate of leukopenia was higher in the ambulatory chemotherapy group (49.28%) than routine chemotherapy group (35.0%)(*P* < 0.01). Days of hospitalization and medical costs were less in the ambulatory chemotherapy group (4.48 d *vs* 14.04 d, ￥6 911.32 *vs* ￥14 623.59, all *P* < 0.01).

**Conclusion:**

Ambulatory chemotherapy is a favorable way to promote.

肺癌发病率和死亡率在世界范围内呈不断上升的趋势，在中国的部分发达地区，肺癌发病率已经位居各种肿瘤的首位^[1]^，如何尽可能满足患者的就医需求是临床医师面临的一个难题，在医院床位数量相对固定情况下，我们需要探索新的医疗模式，以进一步解决患者住院难问题。日间化疗是指肿瘤患者白天住院化疗，晚上回家休息的化疗模式，由于其有效缩短了住院时间并节省了住院费用，已经成为欧美国家最普遍的肿瘤化疗模式。2008年7月上海市胸科医院开设肺癌日间化疗病房，明显加快了周转，提高了出入院率，降低了住院费用，解决患者住院难问题。经过一年多的探索，逐渐形成了一套相对成熟的日间化疗管理模式。由于日间化疗较常规住院化疗具有一定的特殊性，深入了解该治疗模式的安全性问题有助于该模式的改进和推广，故自肺癌日间化疗开始之初即对其安全性进行了回顾性分析。

## 1 资料和方法

### 1.1 临床资料

自2008年10月1日-2009年4月30日，对入住肺部肿瘤中心的患者详细记录日间化疗和普通化疗病房患者的一般特征，包括性别、年龄、PS评分、化疗方案、化疗次数，同时记录2种化疗模式的不良反应，包括常见的胃肠道反应、骨髓抑制、皮肤反应等，统计化疗相关费用、平均住院天数等，两组进行比较。

### 1.2 日间化疗病房患者纳入标准

① 细胞学或组织学证实的非小细胞肺癌或小细胞肺癌；②患者既往进行过至少1次化疗；③此前化疗期间无严重不良反应；④患者一般状况良好，PS评分为0分-1分。

日间化疗患者排除标准：①PS评分在2分或以上；②既往化疗出现Ⅲ度以上胃肠道反应和骨髓抑制、急性过敏反应等严重不良事件；③严重影响化疗的伴发病或禁忌症。

### 1.3 普通化疗病房患者纳入标准

① 细胞学或组织学证实的非小细胞肺癌或小细胞肺癌；②患者一般状况良好，PS < 3分。

普通化疗患者排除标准：①PS评分在2分或以上；②既往化疗出现致命性严重不良事件；③严重影响化疗的伴发病或禁忌症。

### 1.4 毒副反应

按照美国NCI制定的毒副反应标准（CTC第3版）评价毒副反应，分为0-4共5个等级。

### 1.5 数据分析

采用SPSS 10.0统计学软件进行分析，计数资料采用卡方检验比较组间差异，计量资料的比较采用*t*检验，*P* < 0.05为差异有统计学意义。

## 2 结果

### 2.1 两组患者一般特征分析

两组患者年龄、性别、分期、接受手术比例等均无统计学差异。日间化疗患者的PS评分较普通化疗组为佳（*P* < 0.01），普通化疗组中初次化疗者较日间组为多（20.8% *vs* 0%, *P* < 0.01）（表 1）。

**1:** 日间化疗与普通化疗组间一般特征的比较 Comparison between ambulatory chemotherapy group and routine chemotherapy group

	Ambulatory chemotherapy group (*n*=416) *n* (%)	Routine chemotherapy group (*n*=1 157) *n* (%)	*P*
Gender (male/female)	266 (63.9)/150 (36.1)	779 (67.3)/378 (22.7)	0.57
Age	59.2±8.1	57.2±8.7	0.78
Adjuvant/no adjuvant	118 (19.5)/298 (80.5)	315 (27.2)/842 (62.8)	0.74
PS score (0-1/≥2)	416 (100)/0 (0)	897 (77.5)/260 (22.5)	< 0.01
NSCLC/SCLC	377 (90.6)/39 (9.4)	1035 (89.5)/122 (10.5)	0.87
Former chemotherapy (0/1/≥2)	0 (0)/92 (22.1)/324 (77.9)	241 (20.8)/297(25.7)/619 (53.5)	< 0.01
NSCLC: non-small cell lung cancer; SCLC: small cell lung cancer.			

### 2.2 两组化疗方案的比较

两组化疗方案均以含铂方案为主（*P* > 0.05）；亚组分析显示两者采用的含铂两药方案存在差异，日间化疗卡铂的使用比例较普通化疗组高（46.9% *vs* 32.8%），普通化疗组顺铂使用比例高于日间化疗（43.0% *vs* 24.7%, *P* < 0.01）（表 2）。

**2:** 日间化疗与普通化疗组间主要化疗方案及比较 Comparison of main chemotherapy regimens between ambulatory chemotherapy group and routine chemotherapy group

	Ambulatory chemotherapy group (*n*=416) *n* (%)	Routine chemotherapy group (*n*=1 157) *n* (%)	*P*
Non-platinum regimen	101 (24.3)	242 (20.9)	0.36
Platinum-based regimen	315 (51.7)	915(79.1)	
Three drugs	10(2.4)	25 (2.2)	0.92
Two drugs	305 (73.3)	890 (76.9)	< 0.01
Carboplatin	195 (46.9)	380 (32.8)	
Cisplatin	103 (24.7)	498 (43.0)	
Other platinum	7(1.7)	12(1.0)	

### 2.3 两组间主要化疗毒副反应比较

日间化疗组与普通化疗组相比不良反应大致相同，日间化疗和普通化疗组主要区别在于日间化疗组胃肠道反应较普通化疗组显著降低，但血液学毒性相应增加（表 3）。

**3:** 日间化疗与普通化疗组间化疗相关毒副反应（≥Ⅱ度）的比较 Comparison of toxicity (≥Ⅱ) between ambulatory chemotherapy group and routine chemotherapy group

	Ambulatory chemotherapy group (*n*=416) *n* (%)	Routine chemotherapy group (*n*=1 157) *n*(%)	*P*
Non-hematologic			
Vomiting	91 (21.88)	450 (38.89)	< 0.01
Diarrhea	15(3.61)	39 (3.37)	0.83
Fever	35(8.41)	80 (6.91)	0.35
Myalgia	41 (9.86)	102 (8.82)	0.56
Anaphylactic reaction	8(1.92)	20(1.73)	0.80
Heart toxicity	3 (0.72)	12(1.04)	0.57
Phlebitis	1 (0.24)	3 (0.26)	0.62
Dysfunction of liver function	5(1.20)	15(1.30)	0.88
Alopecia	62 (14.90)	170 (14.69)	0.93
Ucosal Inflammation	15(3.61)	46 (3.98)	0.75
Rash	21 (5.05)	62 (5.36)	0.82
Hematologic			
Anemia	32 (7.69)	83 (7.17)	0.75
Leucopenia	205 (49.28)	405 (35.00)	< 0.01
Neutropenia	105(25.24)	228(19.71)	0.06
Thrombocytopenia	51 (12.26)	121 (10.46)	0.37

### 2.4 两组间医疗费用比较

日间化疗与普通化疗相比，住院天数显著降低，有效节省了医疗费用（表 4）。

**4:** 日间化疗与普通化疗组间人均费用的比较（元） Medical cost between ambulatory chemotherapy group and routine chemotherapy group (￥)

	Ambulatory chemotherapy group (*n*=416)	Routine chemotherapy group (*n*=1 157)	*P*
Drug cost	6 204.85^*^	11 802.07	< 0.01
Non-drug cost	706.47	2 821.52	< 0.01
Total cost	6 911.32	14 623.59	< 0.01
Days of hospitalization	4.48	14.04	< 0.01
^*^including outpatient cost.			

## 3 讨论

日间化疗模式平均住院时间大大缩短，住院费用降低，另外可以使部分不适合日间化疗的危重患者接受住院观察，降低了医疗风险，使住院病床得到最大化使用。因此日间化疗已经成为目前国际上一种比较通行的用以降低医疗费用、提高医疗资源使用效率的做法，在美国、英国、澳大利亚、新加坡等^[2, 3]^发达国家广泛开展。国内部分医院也相继开展了门诊化疗和日间化疗等^[4, 5]^治疗模式，但专门肺癌日间化疗病房尚未开展，上海胸科医院肺部肿瘤中心沿用国外模式，结合中国国情，率先开展肺癌日间化疗病房，即患者采用白天住院化疗，化疗结束回家休息的方式，大大降低了住院天数，节省了医疗开支，同时由于患者得到充分及时的治疗，享有更好的休息、营养保证使得日间化疗的疗效得到保证。通过对2008年10月-2009年4月416人次日间化疗的分析表明，日间化疗患者除白细胞降低外其它主要毒副反应并未明显增加，其疗效和安全性在可控范围内。

涉及日间化疗病人不安全因素主要包括：主管医生分散、化疗药物繁杂、患者病情较重、整体护理实施困难等。Gandhi等^[6]^对10 112例日间化疗进行分析，医疗差错的发生率为3%，包括2%可能造成损害的差错，尽管发生率相当低，但可能对患者造成严重伤害。针对可能发生的不良反应，我院日间化疗病房采取了一些措施：委派专门的医生和护理人员管理日间化疗病房，做到专人专职，同时制订了严格的纳入和排除标准。统计数据表明，416例患者急性不良反应共发生7例（包括3例心脏毒性反应，1例急性过敏反应，1例药物外渗导致的静脉炎症），急性不良反应发生率为1.20%，与国外报道相似。

不同化疗方案产生的不良反应不同^[7]^。恶心、呕吐等胃肠道反应是最常见的化疗不良反应，顺铂是目前致吐作用最强的药物之一，本中心研究表明，由于日间化疗采用含卡铂方案多于顺铂，因此急性呕吐反应较住院病人少（*P* < 0.01），对于另外一种常见的血液学毒性方面，日间治疗组则多于住院病人（*P* < 0.01），原因可能由于卡铂的骨髓抑制作用较顺铂为重，另外，日间化疗患者均为既往有化疗史的患者，其累积化疗毒性也是导致骨髓抑制的重要原因。因此，对于日间化疗的患者，应该更加密切注意药物引起的骨髓抑制，日间化疗患者中建立回访制度，专人对化疗间期患者行电话随访，指导患者进行血象监测及其它不良反应处理。

应对化疗过程中及化疗后可能出现的不良反应（adverse events, AE）及严重不良反应（serious adverse events, SAE），建立和完善了肺癌化疗不良反应标准处理流程（standard operation procedure, SOP），包括不良事件及严重不良事件处理SOP、粒细胞减少病人护理SOP、化疗药的配置SOP、抢救车管理SOP、心电监护仪操作SOP等30项不良反应处理规范。同时建立日间化疗24 h随访呼叫系统，保证日间化疗患者出现任何原因的不良反应能够得到及时指导，与相关科室配合，建立急诊绿色通道，对严重不良事件做到随时处理。这些措施有效保证了病人安全性。

日间化疗所有工作人员均能熟练按照SOP流程处理常见严重不良事件。统计表明，日间化疗的患者总体不良反应发生率和住院病人相当（*P* > 0.05），416例患者未发生危及患者生命的严重不良事件发生。

通过比较发现，日间化疗大大降低了住院患者的住院天数，患者平均住院天数为4.48天，而普通住院患者天数为14.04天。由于住院天数明显缩短，节省了床位、护理及辅助用药等不必要的医疗开支，因此极大节省了患者的医疗支出，日间患者平均费用为6 911.32元，远低于住院患者14 623.59元的平均费用（*P* < 0.01）。需要说明的是本统计数据中日间化疗组约25%的化疗方案分为2次住院用药（如GP方案为第1天和第8天化疗），统计时如将同一周期的2次住院化疗的费用或住院时间合并，所得每周期化疗的费用略高于本文统计表中费用，住院天数也高于本文统计值；但仍然显著低于普通住院的总费用和住院天数。

日间化疗在中国还属于新生事物，尽管发达国家有些经验可以借鉴，但国情不同，采用的医疗模式有差别，因此我们需要探索有中国特色的医疗模式，肺癌日间化疗病房的成立和运行的研究表明，日间化疗在当前医疗资源紧张的情况下有效降低了医疗成本，提高医疗资源使用效率，而且并不增加患者的严重不良反应事件。未来还需要更多研究证实该模式在中国的可行性和安全性。
